# Botulinum Toxin Treatment Can Enlarge Eye Appearance in Asian Patients and Improves Social and Emotional Attributes

**DOI:** 10.3390/toxins18030145

**Published:** 2026-03-15

**Authors:** Maurício de Maio, Kiyoko Kato, Momoko Sato, Yuki Horiuchi, Takuya Toyama, Akiko Imaizumi, Hidenori Ishii

**Affiliations:** 1 MD Codes Institute, Sao Paulo 04528-133, SP, Brazil; 2 Azabu Beauty Clinic Medical Corporation, Tokyo 106-0047, Japan; kato@abcsalon.co.jp; 3 Injection Lab, Osaka 541-8513, Japan; 4 Akihabara Skin Clinic, Tokyo 101-0021, Japan; 5 Toyama Plastic & Cosmetic Surgery, Okinawa 900-0015, Japan; 6 Imaizumi Skin Clinic, Tokyo 106-0032, Japan; 7 Otsuka Cosmetic & Plastic Surgery, Tokyo 101-8535, Japan

**Keywords:** botulinum neurotoxin type A, emotional attributes, eye size, myomodulation, onabotulinumtoxinA, social attributes

## Abstract

Aesthetic patients in East Asia are commonly concerned about small apparent eye size. Simultaneous treatment of the glabellar and lateral canthal areas with botulinum neurotoxin has potential to provide improvements. This case series evaluated changes in eye size following treatment of these two areas using standard on-label doses of onabotulinumtoxinA in patients from Japan or China. Outcomes were assessed based on standardised frontal photographs taken before and after treatment (at rest, maximum smile, and maximum frowning). Changes in eye size were examined using a 4-point Likert scale, as evaluated by three independent groups: six injectors; six non-injecting observers; and treated patients. Furthermore, improvements in overall facial impression were analysed using two established tools: ‘emotional attributes’ and ‘social attributes’. Twenty East Asian subjects were included (n = 17 women; mean age: 37.5 ± 6.4 years). The majority of evaluators in all three groups rated patients’ eye size as ‘significantly’ or ‘mildly’ improved post-treatment, whether assessed at rest, when smiling, or during frowning. Furthermore, almost all evaluators noted improvements in one or more emotional and social attributes. This approach has significant potential as a culturally adapted aesthetic technique for improving eye size in East Asian patients. Larger multicentre studies are warranted.

## 1. Introduction

It is well established that humans form initial impressions of other people’s personalities within the first few seconds of meeting them—largely based on the appearance of their face [1,2]. The eyes may play a particularly important role in this process. Furthermore, across ethnic groups and cultures, larger eyes are typically associated with greater attractiveness [3,4,5,6]. Compared to Western populations, East Asians generally have smaller palpebral fissures [7]. This is reflected in the popularity of aesthetic procedures designed to enlarge the eyes, with eyelid surgery ranking as the most frequently performed cosmetic surgical procedure in Japan in 2024 [8].

While surgery remains a key option for increasing eye size, nonsurgical modalities are also available and offer potential advantages with regard to the non-permanence of results, decreased downtime, and reduced safety concerns [9,10]. Botulinum neurotoxin type A (BoNTA) formulations, such as onabotulinumtoxinA, have long been used to treat dynamic rhytides. In our clinical experience, simultaneous treatment of the glabellar and lateral canthal areas with BoNTA can also be used to enlarge the appearance of the eyes in East Asian patients.

We therefore performed a pilot review of a series of cases to assess this possibility and examine whether such effects persist during animation (smiling and frowning). In addition, the contribution of these effects to improving the overall impression of the face was evaluated using two established tools—the ‘emotional attributes’ and the ‘social attributes’ [11,12,13]. Each of these incorporates eight common characteristics by which the impact of aesthetic treatment of the face may be perceived (Table 1).

## 2. Results

A total of 20 East Asian patients were included in the analysis (17 Japanese and 3 Chinese). Of these, 17 (85%) were women and 3 (15%) were men, and the mean age was 37.5 ± 6.4 years (range: 28–49 years). All were injected with onabotulinumtoxinA using the standard on-label dose and administration pattern for aesthetic treatment of the glabella (20 U) and lateral canthal areas (12 U per side).

Six injectors each independently assessed pre- and post-treatment photographs for all 20 patients, thus yielding a total of 120 evaluations. Similarly, six observers—all of whom were nurses or clinic staff—independently assessed the same images (thus N = 120 in total). In addition, 17 of the patients, excluding the 3 Chinese individuals who were unavailable for follow-up, assessed their own images (thus N = 17 in total).

**Figure 1:**
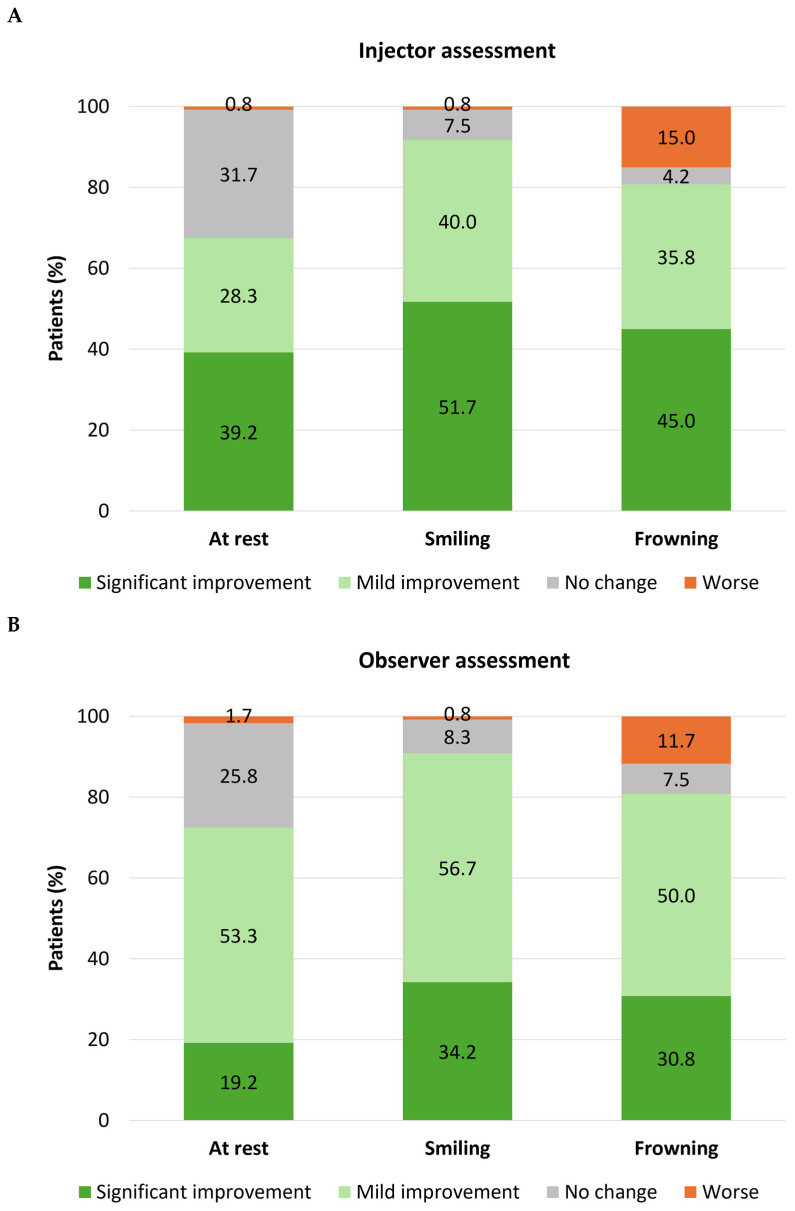

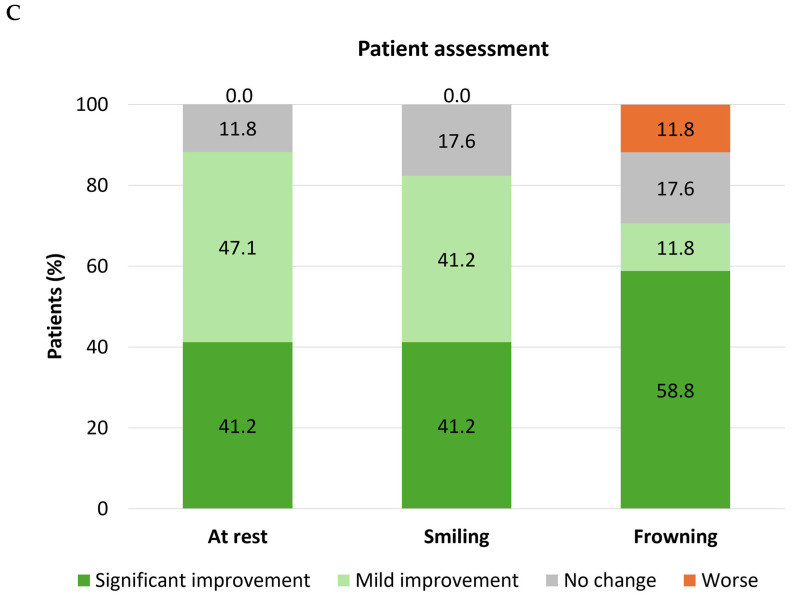
Change (increase) in eye size following treatment with onabotulinumtoxinA. Eye size in post-treatment photographs was assessed relative to pre-treatment images by injectors (N = 120; **part A**), observers (nurses/clinic staff; N = 120; **part B**), and patients (N = 17; **part C**).

Based on a 4-point Likert scale, injectors rated most patients’ eye size as ‘significantly’ or ‘mildly’ improved post-treatment, whether assessed at rest (n = 81; 67.5%), at maximum smile (n = 110; 91.7%), or at maximum frown (n = 97; 80.8%) (Figure 1A). Observers rated eye size as significantly or mildly improved in similar numbers of cases, evaluated at rest (n = 87; 72.5%), at maximum smile (n = 109; 90.8%), or at maximum frown (n = 97; 80.8%) (Figure 1B). Furthermore, a majority of patients also rated their own eye size as significantly or mildly improved at rest (n = 15; 88.2%), at maximum smile (n = 14; 82.4%), or at maximum frown (n = 12; 70.6%) (Figure 1C). Few injectors, observers, or patients rated eye size as ‘worse’ post-treatment in images taken at rest or when smiling (<2% for all groups of assessors). However, when evaluated during frowning, some injectors (n = 18; 15.0%), observers (n = 14; 11.7%), and patients (n = 2; 11.8%) said that eye size was worse.

**Figure 2:**
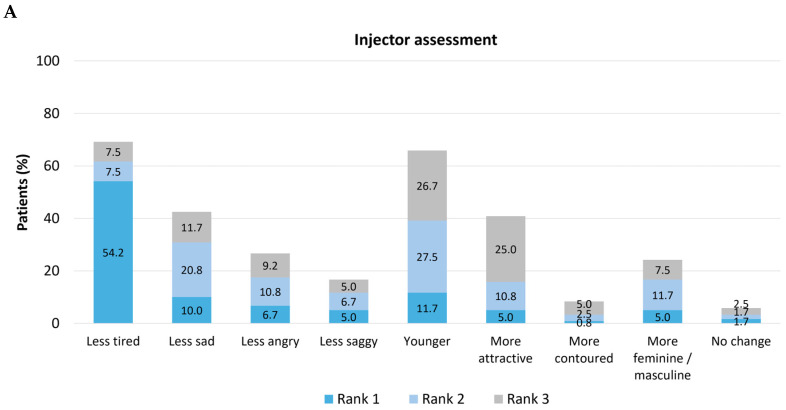

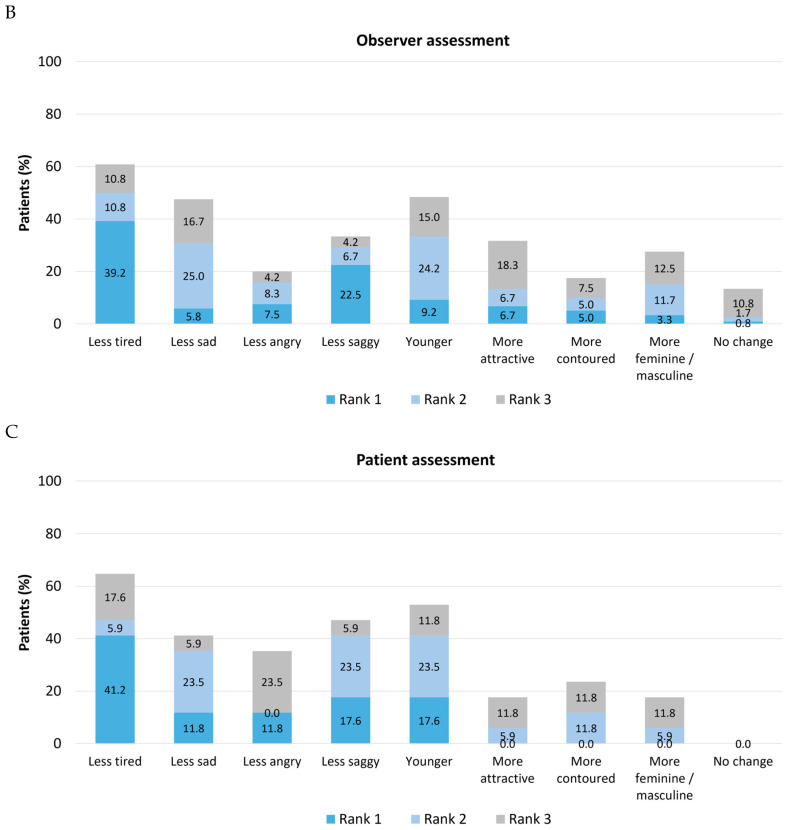
Assessment of emotional attributes following treatment with onabotulinumtoxinA. Emotional attributes were assessed and ranked in post-treatment photographs relative to pre-treatment images by injectors (N = 120; **part A**), observers (nurses/clinic staff; N = 120; **part B**), and patients (N = 17; **part C**).

All of the evaluators were then asked to consider emotional attributes in the post-treatment images and rank the top three showing the most improvement (Figure 2). For all of the evaluator groups, the two emotional attributes most commonly placed in the top three were appearing ‘less tired’ (injectors, n = 83, 69.2%; observers, n = 73, 60.8%; patients, n = 11, 64.7%) and appearing ‘younger’ (injectors, n = 79, 65.8%; observers, n = 58, 48.3%; patients, n = 9, 52.9%). Few physicians (n = 2, 1.7%), observers (n = 1, 0.8%), or patients (n = 0, 0%) put ‘no change’ in rank 1, suggesting that there was almost always an improvement in at least one emotional attribute.

**Figure 3:**
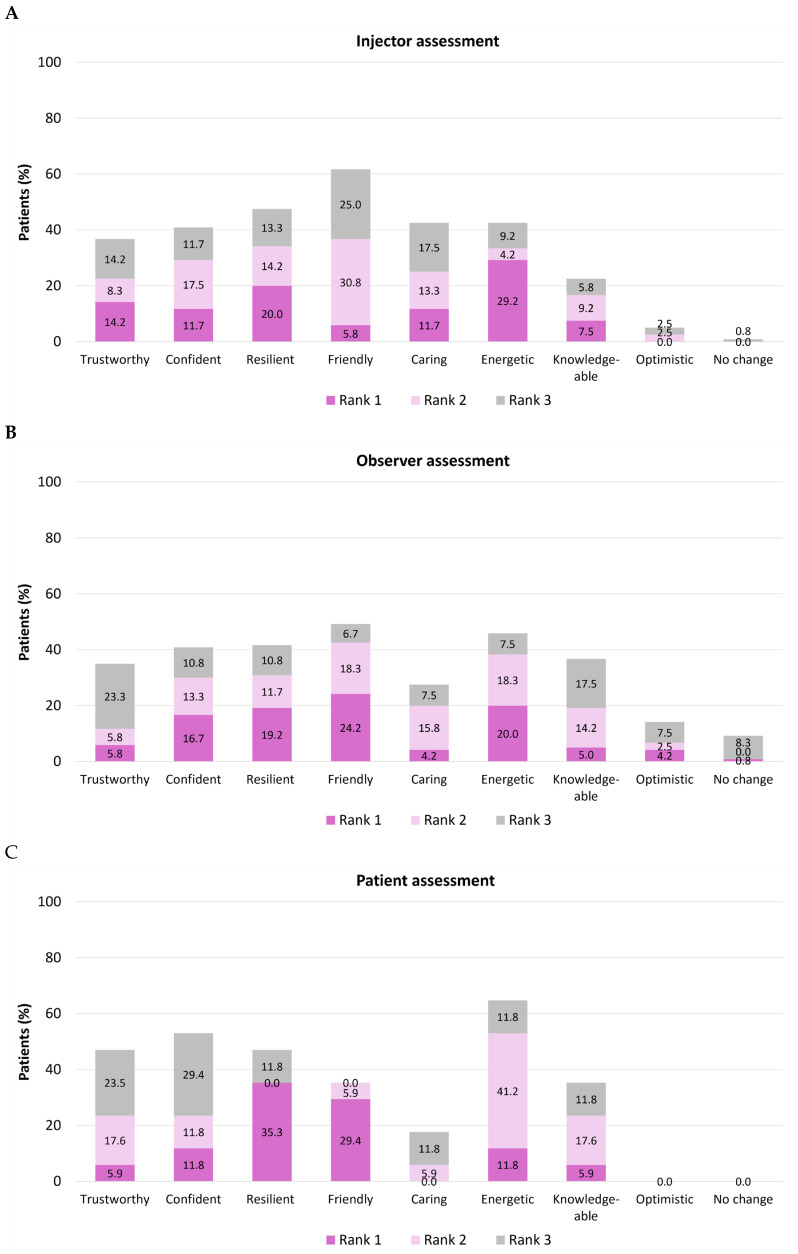
Assessment of social attributes following treatment with onabotulinumtoxinA. Social attributes were assessed and ranked in post-treatment photographs relative to pre-treatment images by injectors (N = 120; **part A**), observers (nurses/clinic staff; N = 120; **part B**), and patients (N = 17; **part C**).

Evaluators were also asked to assess social attributes in the post-treatment images and rank the three with the most improvement (Figure 3). Among injectors, the two social attributes most commonly ranked in the top three were appearing ‘more friendly’ (n = 74, 61.7%) and appearing ‘more resilient’ (n = 57, 47.5%). Among observers, the two most commonly selected were ‘more friendly’ (n = 59, 49.2%) and ‘more energetic’ (n = 55, 45.8%). Patients themselves most frequently rated their own appearance as ‘more energetic’ (n = 11, 64.7%) and ‘more confident’ (n = 9, 52.9%). Few physicians (n = 0, 0%), observers (n = 1, 0.8%), or patients (n = 0, 0%) placed ‘no change’ in rank 1, suggesting that there was almost always an improvement in at least one social attribute.

**Figure 4:**
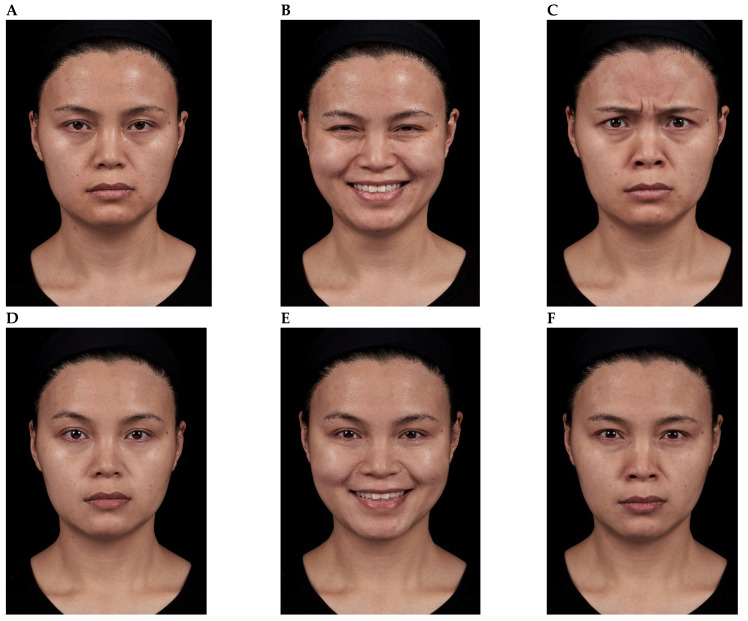
Impact of onabotulinumtoxinA treatment of the glabellar and lateral canthal regions. A 36-year-old female shown before treatment (at rest, maximum smile, maximum frown, **parts A**–**C**) and 3 days after treatment of the glabellar and lateral canthal areas with onabotulinumtoxinA (at rest, maximum smile, maximum frown, **parts D**–**F**).

**Figure 5:**
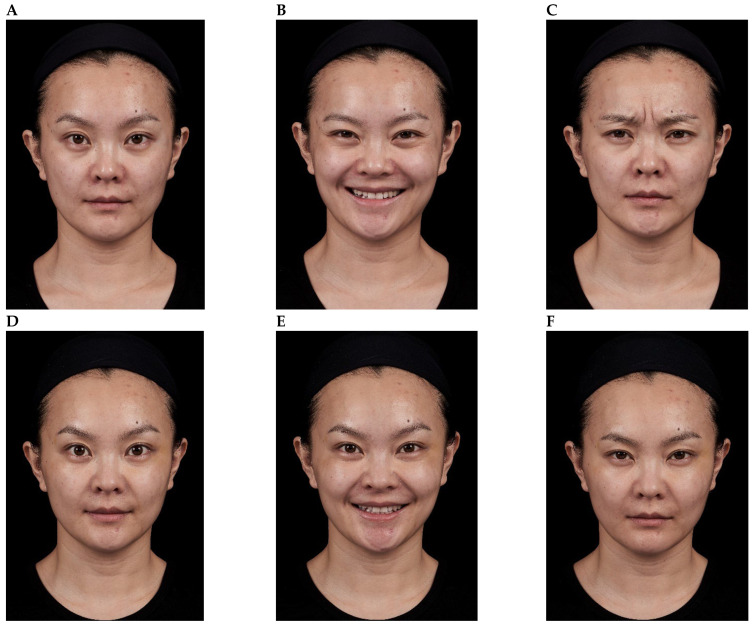
OnabotulinumtoxinA treatment of the glabellar and lateral canthal regions. A 32-year-old female shown before treatment (at rest, maximum smile, maximum frown, **parts A**–**C**) and 3 days after treatment of the glabellar and lateral canthal areas with onabotulinumtoxinA (at rest, maximum smile, maximum frown, **parts D**–**F**).

**Figure 6:**
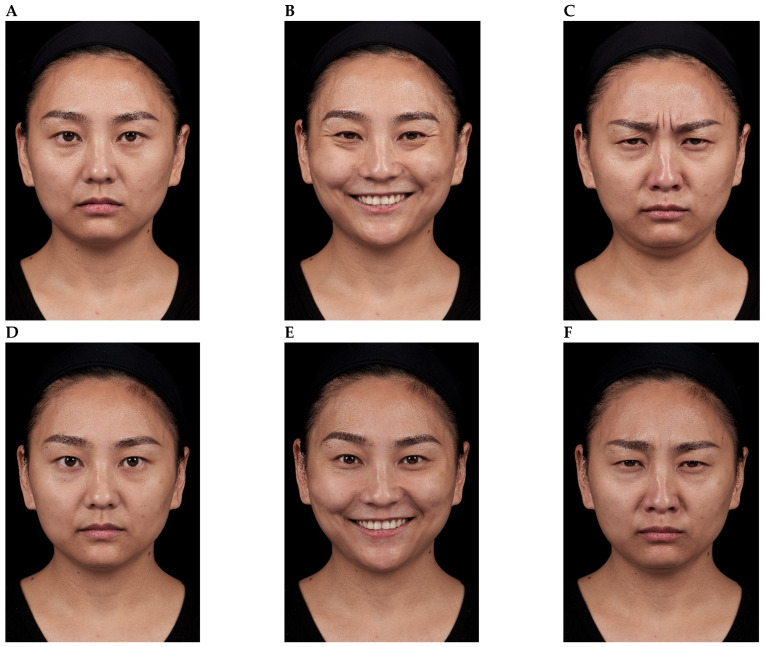
OnabotulinumtoxinA treatment of the glabellar and lateral canthal regions. A 34-year-old female shown before treatment (at rest, maximum smile, maximum frown, **parts A**–**C**) and 3 days after treatment of the glabellar and lateral canthal areas with onabotulinumtoxinA (at rest, maximum smile, maximum frown, **parts D**–**F**).

**Figure 7:**
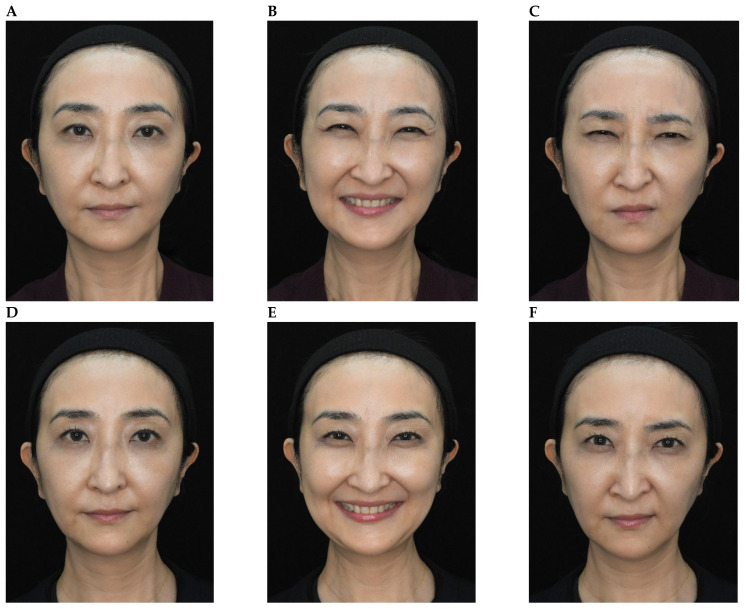
OnabotulinumtoxinA treatment of the glabellar and lateral canthal regions. A 52-year-old female shown before treatment (at rest, maximum smile, maximum frown, **parts A**–**C**) and 5 days after treatment of the glabellar and lateral canthal areas with onabotulinumtoxinA (at rest, maximum smile, maximum frown, **parts D**–**F**).

**Figure 8:**
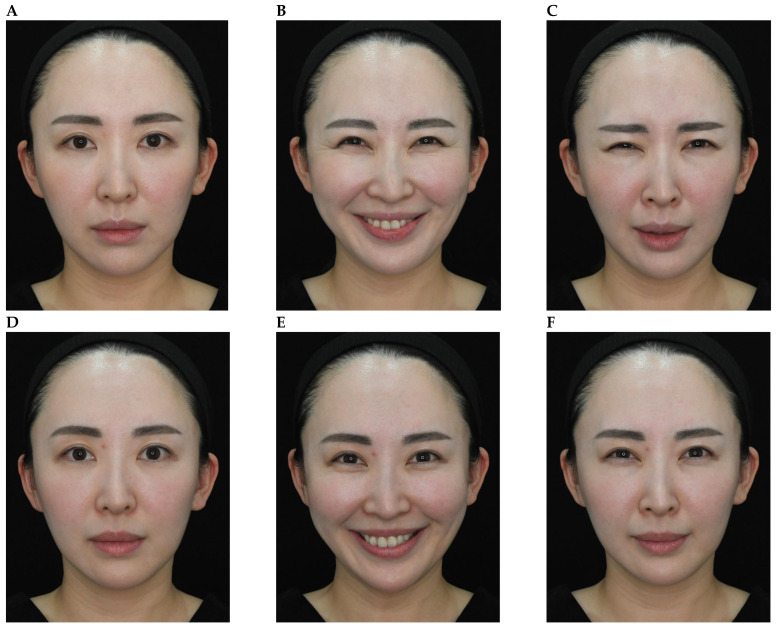
OnabotulinumtoxinA treatment of the glabellar and lateral canthal regions. A 39-year-old female shown before treatment (at rest, maximum smile, maximum frown, **parts A**–**C**) and 5 days after treatment of the glabellar and lateral canthal areas with onabotulinumtoxinA (at rest, maximum smile, maximum frown, **parts D**–**F**).

Representative results are shown in Figure 4, Figure 5, Figure 6, Figure 7 and Figure 8. No adverse events were reported for any of the included patients.

## 3. Discussion

This case series demonstrated improvements in eye size following onabotulinumtoxinA treatment of the glabellar and lateral canthal areas in a group of East Asian subjects—as assessed by injectors, non-injecting clinical observers, and patients themselves. There were also consistent improvements in how their faces were perceived, evaluated using emotional and social attributes [11,12,13]. To the best of our knowledge, this is the first case series to systematically demonstrate the impact of BoNTA treatment of these two facial areas on both eye size and overall facial impression.

The results have important clinical implications given the well-established association between eye appearance and perceived attractiveness [3,4,5,6]. Indeed, a recent Chinese study found that the eyes were the single most significant predictor of attractiveness across all facial features assessed [6]. The value of nonsurgical ‘opening’ of the eyes may be particularly noteworthy in East Asian populations, where patients often present with heavier upper eyelids and smaller eyes compared with Caucasians, and where there is a strong cultural emphasis on larger eyes as a desirable aesthetic ideal. Although BoNTA injections into the lower eyelid have previously been shown to effectively widen the eyes [14,15], paralysis of orbicularis oculi in the lower eyelid region is often less preferred among East Asian patients—for whom the pretarsal roll is usually considered as a positive aesthetic feature [16,17,18]. Thus, the present method offers a valuable, culturally adapted alternative.

**Figure 9:**
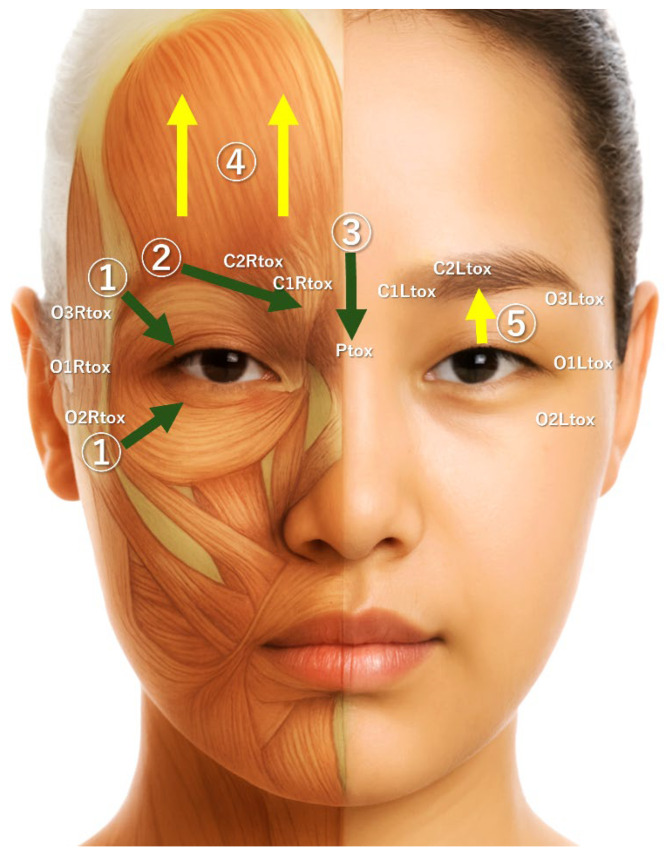
Putative mechanisms of eye enlargement. Relevant MD DYNA Codes for BoNTA injection (4 U per site) are based on: C1tox, medial corrugator; C2tox, lateral corrugator; Ptox, procerus; O1tox, central lateral orbital; O2tox, lower lateral orbital; and O3tox, upper lateral orbital. ‘R’ and ‘L’ denote the right and left sides of the face, respectively. Green downwards arrows indicate the blocking of muscle function by BoNTA: ① orbicularis oculi; ② corrugator supercilii; ③ procerus. Yellow upward arrows indicate enhancement of muscle activity: ④ frontalis; ⑤ levator palpebrae superioris. BoNTA, botulinum neurotoxin type A.

The effects observed may be explained through three possible underlying mechanisms: (1) glabellar injections reduced the activity of brow depressor muscles (corrugator, procerus, and orbicularis oculi), thereby enhancing the action of the frontalis muscle and elevating the eyebrows; (2) paralysis of the lateral orbicularis oculi reduced downward traction on the upper eyelid, preventing excessive eyelid hooding during smiling; and (3) weakening of orbicularis oculi activity resulted in functional enhancement of the levator palpebrae superioris (Figure 9) [19,20].

The first author has previously proposed that modulation of muscle activity and facial expression can be achieved through two distinct approaches: ‘chemical myomodulation’ and ‘mechanical myomodulation’ [21]. Chemical myomodulation primarily involves the use of BoNTA to suppress muscle activity, thereby reducing dynamic facial lines and improving the morphological changes caused by muscular hyperactivity. By contrast, mechanical myomodulation mainly utilises injectable fillers, such as hyaluronic acid (HA), to modulate muscle movement through physical and structural alterations. The treatment approaches for individual muscles have been systematised and defined as the MD DYNA Codes, a symbolic coding system representing the targeted muscles and the corresponding methods of modulation [22]. The concept of mechanical myomodulation has been further elucidated in several additional studies [23,24,25,26,27]. The present case series focused on morphological changes induced by chemical myomodulation alone—applying the MD DYNA Codes known as ‘Ctox’ (BoNTA injection into corrugator supercilia), ‘Ptox’ (BoNTA injection into procerus), and ‘Otox’ (BoNTA injection into orbicularis oculi) (Figure 9).

**Figure 10:**
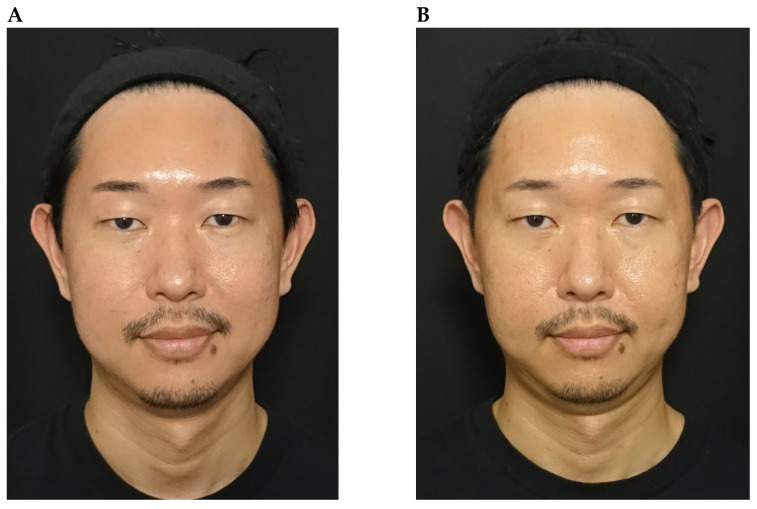
Reduced impact of onabotulinumtoxinA treatment on eye size in a patient with a single eyelid. A 37-year-old male shown at rest before treatment (**part A**) and 26 days after treatment of the glabellar and lateral canthal areas with onabotulinumtoxinA (**part B**).

**Figure 11:**
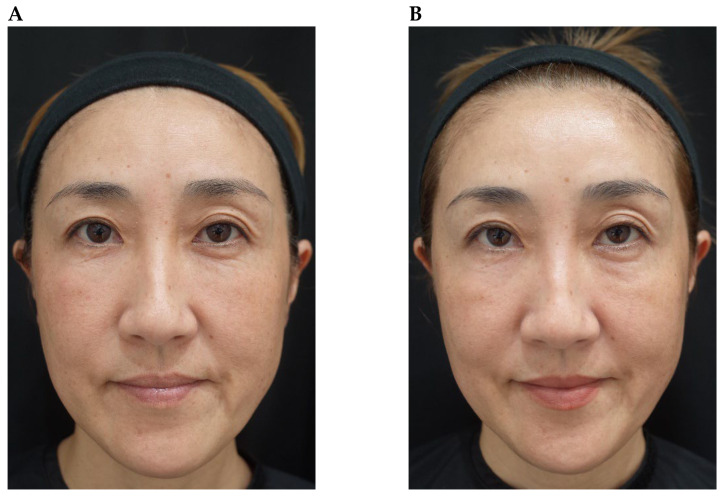
Reduced impact of onabotulinumtoxinA treatment on eye size in a patient with marked upper eyelid volume loss. A 49-year-old female shown at rest before treatment (**part A**) and 21 days after treatment of the glabellar and lateral canthal areas with onabotulinumtoxinA (**part B**).

**Figure 12:**
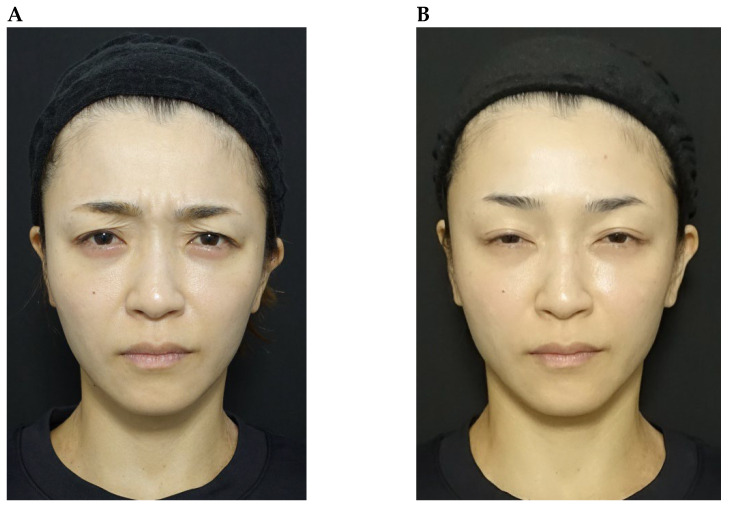
Reduced impact of onabotulinumtoxinA treatment on eye size in a patient whose palpebral aperture decreased in size during frowning. A 37-year-old female shown during frowning before treatment (**part A**) and 26 days after treatment of the glabellar and lateral canthal areas with onabotulinumtoxinA (**part B**).

Although favourable outcomes were observed in the majority of cases, the treatment effect was limited in three subjects: (a) a patient with a single eyelid (monolid; Figure 10); (b) an individual with marked upper eyelid volume loss (Figure 11); and (c) a patient whose palpebral aperture decreased in size during frowning (Figure 12). Functionally, the orbicularis oculi acts antagonistically to the levator palpebrae superioris during eyelid movement. However, in patients like case (a) with a single eyelid, changes in opening are less visually apparent, which may explain the limited perceptible effect despite functional modulation [20]. Some single-eyelid patients improve with BoNTA treatment alone but others do not; in the latter cases, surgical intervention may be discussed as an alternative. Cases (b) and (c) reflect the inherent limitations of chemical myomodulation, which can potentially be addressed through the combined use of mechanical myomodulation. In the patient with marked upper eyelid volume loss, this was accompanied by eyelid ptosis. In such circumstances, functional enhancement of the levator palpebrae superioris through chemical myomodulation with BoNTA alone may be insufficient to overcome the underlying structural deficit, and mechanical myomodulation using HA fillers might therefore be needed to restore structural support. With regard to the patient whose palpebral aperture decreased in size during frowning, weakening of the orbital portion of orbicularis oculi could have resulted in compensatory recruitment of the palpebral or pretarsal portion. However, objective evidence demonstrating a true increase in muscle strength remains limited, and this compensatory mechanism may contribute to the reduced treatment effect observed.

Notably, even patients with apparent worsening of eye size on frowning showed improvements in how their face was perceived based on emotional and social attributes. Moreover, it is reassuring that the two emotional attributes most commonly cited as being improved were the same among injectors, observers, and patients—appearing ‘less tired’ and ‘younger’—suggesting that these effects were consistently achieved. Previous studies of the emotional and social attributes were based on full-face treatment using HA fillers [12,13]. Hence, this is the first demonstration of their value in assessing the impact of BoNTA injections—and also the first time they have been used to examine targeted treatment with a specific clinical goal (opening of the eye aperture) rather than full-face aesthetic improvement.

Clinicians in East Asia often administer smaller doses of BoNTA for aesthetic indications in the upper third of the face compared with western countries. Reasons may include lower muscle mass and reduced hyperdynamic activity among Asian patients [28], as well as a perception that results may be more ‘natural looking’ with a lower dose. However, in the present series, patients were treated with the maximum recommended dose, as per product labelling (20 U in the glabella, and 12 U per side in the lateral canthal area). The demonstration that these doses can meaningfully increase the apparent size of the eye—beyond the well-known effects on rhytides—may provide a rationale for using the maximum recommended dose more often in routine practice. Further studies are required to assess optimal strategies.

Irrespective of the dose used, proper injection technique is central to positive outcomes. In particular, it is essential that practitioners recognise individual anatomical variations in muscle insertions, use appropriate BoNTA concentrations, and administer injections precisely into the intended muscles. We recommend the following clinical precautions: performance of dynamic assessment before injection; identification of corrugator supercilii muscle insertions by asking the patient to frown strongly; avoidance of injections medial to the orbital rim; prevention of diffusion towards the levator palpebrae superioris; and, when injecting the procerus, lower placement of injections to avoid overlap with the frontalis and minimise unintended spread.

We must acknowledge the limitations of the current work. In particular, it was a small pilot case series with a retrospective design and no control group. Multiple evaluators assessed outcomes, thus greatly enriching the size of the dataset, but it would nonetheless be valuable to assess the impact of this approach on eye size in a larger cohort of patients using a prospective, controlled study design. In addition, size assessments were subjective, and quantitative measurements were lacking. However, subjective, perception-based outcomes are commonly used in aesthetic medicine and the analyses made in the present work were structured and blinded. We did attempt quantitative measurements using ImageJ software (version 2) but differences were difficult to demonstrate—owing to the subtlety of the changes, variability with animation, and the interference of eyelashes making precise measurement challenging—and that approach was therefore abandoned. Previous studies have also noted the difficulty of quantifying eye changes after BoNTA treatment [14,15]. In the future, AI tools may allow for such analyses. Assessments in this study were limited to eye *size* and it would be valuable also to examine the effect of BoNTA treatment on eye *shape.* In addition, it would be beneficial to incorporate other validated patient-reported outcome tools, such as FACE-Q. Finally, we acknowledge that diurnal variations in eyelid oedema could have affected perceived eye size, although all pre- and post-treatment photographs were taken during the daytime (and never in the early morning or late evening) so the impact is likely to have been minimal.

## 4. Conclusions

Combined injection of onabotulinumtoxinA into the glabellar and lateral canthal regions enlarged the apparent appearance of the eyes and improved how the face was perceived overall in the majority of East Asian patients included in this study. Effects were evident both at rest and during animation. Even individuals who gained no positive effects on eye size still experienced improvements in emotional and social attributes. Our findings suggest that this approach has significant potential as a culturally adapted aesthetic technique. Larger multicentre studies are warranted.

## 5. Materials and Methods

### 5.1. Study Design and Population

This case series evaluated changes in eye size and perceived emotional and social attributes following aesthetic treatment of the glabellar and lateral canthal areas of the face using standard doses of onabotulinumtoxinA. Included patients were adult females or males from Japan or China requesting improvements in eye size and/or reduction in periocular wrinkles. Individuals with severe eyelid ptosis were excluded.

The study was performed in accordance with the ethical principles outlined in the Declaration of Helsinki. As it was a fully retrospective case series using only existing anonymized data, the requirement for Ethics Committee approval was waived. Written informed consent for treatment and for use of their images for research purposes was obtained from all participants prior to injection.

### 5.2. Treatment

The only approved BoNTA (onabotulinumtoxinA) product in Japan is Botox Vista® Injection 50 U (Allergan, an AbbVie company, Madison, NJ, USA), and all Japanese cases were therefore treated using this formulation, reconstituted with 1.25 mL of 0.9% saline solution per vial. For the Chinese cases, Botox® Cosmetic (Allergan, an AbbVie company; 100 U per vial) was used, reconstituted with 2.5 mL of 0.9% saline solution to ensure equivalent concentration and dosing across all patients. The injection protocol employed the standard on-label doses and administration patterns for aesthetic treatment of the glabella (20 U in total, based on 4 U into each of five sites) and lateral canthal areas (12 U per side, based on 4 U into each of three sites per side) [29].

Injections were performed in clinical settings by one of six experienced injectors (five based in Japan; one based in Brazil), under aseptic conditions and in compliance with established anatomical and safety guidelines. Patients received no other aesthetic treatments during the assessment period of this study.

### 5.3. Data Collection and Assessments

Outcomes were assessed based on standardised photographic documentation, using frontal images taken before and after treatment—at rest, at maximum smile, and during maximum frowning.

All post-treatment photographs were taken between 3 days and 1 month after the injections.

Changes in eye size pre- and post-treatment were evaluated using a 4-point Likert scale: 1, worse; 2, no change; 3, mild improvement; 4, significant improvement.

In addition, the impact of treatment on how the patients were perceived was assessed using eight ‘emotional attributes’ and eight ‘social attributes’. These have been published in full elsewhere [11,12,13]. Briefly, the emotional attributes were used to evaluate whether, in post-treatment photographs, the patient looked: less tired; less saggy; less sad; less angry; younger; more attractive; more contoured; and/or more feminine/masculine. The social attributes were used to assessed whether, post-treatment, the patient looked: more trustworthy; more confident; more resilient; more friendly; more caring; more energetic; more knowledgeable; and/or more optimistic. Comparing pre- and post-injection photographs, evaluators were asked to rank the top three emotional attributes and the top three social attributes that had been improved with treatment. If they did not perceive any such improvements (or fewer than three), they were allowed instead to state that there was ‘no change’.

All of these evaluations were carried out by three separate and independent groups. Group 1 included the six injectors (five from Japan and one from Brazil) who assessed pre- and post-treatment photographs for all of the included patients. Group 2 incorporated six non-injecting evaluators (referred to as ‘observers’) who were nurses or clinic staff (one from each of the centres of the six injectors); they also assessed photographs from all of the included cases. Group 3 incorporated each of the treated patients, except those from China due to unavailability for follow-up assessments; this group viewed only their own photographs and not those of the other patients. All evaluations were performed independently to minimise observational bias.

### 5.4. Statistical Analyses

Descriptive statistics are provided throughout, including frequency and percentage for categorical variables, and mean, standard deviation, and range for continuous variables.

## Figures and Tables

**Table 1:** Emotional and social attributes of the face.

Emotional Attributes	Social Attributes
Look less tired	Look more trustworthy
Look less sad	Look more confident
Look less angry	Look more resilient
Look less saggy	Look more friendly
Look younger	Look more caring
Look more attractive	Look more energetic
Look more contoured	Look more knowledgeable
Look more feminine/masculine	Look more optimistic

Table adapted from references [12] and [13].

## Data Availability

The original contributions presented in this study are included in the article. Further inquiries can be directed to the corresponding author.
